# Self-Reported Dysphagia and Laryngopharyngeal Reflux among a Community-Dwelling Elderly Rural Population

**DOI:** 10.1055/s-0045-1810025

**Published:** 2025-10-09

**Authors:** Giorgos Sideris, Melina Kourklidou, John Plioutas, Ilias Georgantis, Eleni Petridou, Alexander Delides

**Affiliations:** 1 Second Otolaryngology Department, School of Medicine, National and Kapodistrian University of Athens, Attikon University Hospital, Athens, Greece; 2 Department of Hygiene Epidemiology and Medical Statistics, School of Medicine, National and Kapodistrian University of Athens, Athens, Greece

**Keywords:** dysphagia, laryngopharyngeal reflux, epidemiology, elderly

## Abstract

**Introduction:**

There is a lack of community-dwelling population-based examinations of geriatric dysphagia and laryngopharyngeal reflux (LPR).

**Objective:**

To assess the prevalence of geriatric dysphagia and laryngopharyngeal reflux (LPR) in a community-dwelling population from a rural area.

**Methods:**

Volunteers from Velestino, a 4,000-person community, were given the Eating Assessment Tool (EAT-10) and Reflux Symptom Index (RSI) questionnaires at the health center. Demographic, medical, and pharmaceutical histories were recorded.

**Results:**

160 participants (92 females, 68 males) self-presented. Ages were between 65–95 years (Mean 76.04). 27 (16.88%) scored an abnormal EAT-10 and 13 (8.13%) RSI over 13. RSI was statistically correlated with total and positive only EAT-10 scores. Age and gender did not affect EAT-10 or RSI scores. The EAT-10 score decreased with age progression.

**Conclusion:**

This is one of the few studies to investigate the relationship between geriatric dysphagia and LPR in a rural population. A 23.0% prevalence of dysphagia was observed in elderly adults as well as an increase in both EAT-10 and RSI scores with age progression. More studies are needed to explore this field.

## Introduction

Dysphagia, or difficulty swallowing, is a common problem in the elderly. 1 2 Both dysphagic and geriatric patients are malnourished, conditions that lead to sarcopenia and aspiration pneumonia. An increasing number of elderly individuals are hospitalized due to pneumonia and malnourishment. 3 4 Laryngopharyngeal reflux (LPR) is a complex of symptoms and findings attributed to extra-esophageal reflux of gastric juice and is one of the main causes of dysphagia. 5

Various reports exist on the prevalence of dysphagia among the elderly with figures reaching even 63%, but most of them involve either hospitalized patients or institutionalized individuals. 1 6 Many reports on dysphagia within nursing homes also exist, but most of them do not differentiate between otherwise healthy or individuals with co-morbidities. 7

According to studies, there seems to be a worldwide wide diversity of prevalence figures between reports, which confuses those who seek to influence public health policy makers for initiatives towards taking preventive measures for dealing with the problem. 7 On the other hand, over-estimation of the prevalence of dysphagia among the geriatric population might lead to unnecessary increase of healthcare cost since the total elderly population is on the rise. In 2050, elderly Americans, defined as those at least 65 years old, are expected to make up 20% of the total population, a substantial increase from 13% in 2010. Globally, the number of people aged 60 or above is expected to more than double by 2050 and more than triple by 2100. According to the UN, a significant ageing of the population in the next several decades is projected for most regions of the world, starting with Europe where 34% of the population is projected to be over 60 years old by 2050. 8

Thus, the immerging importance of dysphagia as a social, health and economic burden upon countries must be accurately defined and calculated to convince countries and institutions to act preventively on one hand but not overinflate the cost on the other. However, it is reported that sociodemographic groups at higher risk for dysphagia are less likely to receive treatment. Thus, targeted interventions are needed to address barriers to care. 9

Both LPR and dysphagia are prevalent among the elderly, but no community-dwelling population- based studies investigating both entities exist. 10 11

Accurate diagnosis of LPR requires objective pH monitoring but such a testing is not used in everyday practice. 10 Screening for LPR as well as dysphagia is frequently based upon simple to use, validated in many languages, questionnaires such as the Reflux Symptom Index (RSI) and the Eating Assessment Tool (EAT-10), respectively. 5 Both the aforementioned questionnaires have been validated in the authors' language 12 13 and are used among Otolaryngologists for more than a decade 14 in everyday practice to screen patients for LPR and dysphagia and monitor response to treatment. RSI lacks some sensitivity in the elderly population 15 since it seems that these patients tend to lose sensitivity with aging. So it should not be far from true to hypothesize that the actual prevalence of LPR patients in this population would be higher than what will be shown with the RSI.

To calculate the prevalence of self-reported dysphagia and LPR within an otherwise “healthy”, community-dwelling, elderly population we performed a population survey in a Greek rural area.

## Methods

### The “VELESTINO” Study's Sample

Data collection was conducted in the municipality of Velestino, a town in central Greece of about 4,000 inhabitants, during two visits. Attempts were made to contact all individuals of the target group prior to the investigator's arrival through house visits, visits to senior day centers, and contact with hospital administrators and practicing physicians. Individual invitations were sent and local representatives made phone calls to individuals older than 60 years old according to the town's registry.

On two different occasions, investigators (including two certified Otolaryngologists and two senior Otolaryngology residents) visited the local health center. Participants that were voluntarily included in the study self-presented at the health center and after signing an informed consent underwent basic Ear Nose and Throat examination. They were then provided with the Greek version of the EAT-10 and RSI questionnaires. Demographic data, past medical history and history of medications were also recorded.

### Statistical Analysis

Statistical analysis was performed with Graphpad Prism® 6 for Mac OSX. RSI scores > 13 and EAT-10 scores > = 3 were regarded as abnormal. 5 6 7 8 9 10 11 12 13 14 15 16 Confidence Intervals were set at 95%. Pearson correlation coefficients were calculated for comparison between ages, genders and subscores of EAT-10 and RSI.

Linear regression was performed to examine potential relationships between the EAT-10 and RSI outcomes and ages of the participants as well as between EAT-10 and RSI. Mann-Whitney non-parametrical t-tests were used to calculate differences between means of scores. A p value of 0.05 or lower was deemed statistically significant.

## Results

According to the town's registry, 682 individuals were over 65 years of age during the initial selection process of the study and potentially available for participation.

A total of 160 individuals (92 female and 68 male) self- presented to the health center, underwent the examination and completed the questionnaires. Ten of the subjects that were initially presented were under 65 and their scores were excluded from the analyses. Ages were 65 to 95 (Mean 76.04, 95% CI: 75.01-77.07). 27 individuals (16.88%) scored an abnormal EAT-10, and 13 (8.13%) an RSI above 13 ( Table 1 ).

**Table 1:** Self-reported dysphagia and laryngopharyngeal reflux among individuals over 65 years of age

		(N) Mean	CI 95% Lower- Upper	p-value
Age		(160) 76.04	75.01-77.07	
	Male	(68) 78.09	76.59-79.59	**0.0003**
	Female	(92) 74.53	73.19-75.88	
EAT-10 (Total)		(160) 1.56	0.89-2.22	
	Male	(68) 1.21	0.41-2.00	0.1723
	Female	(92) 1.82	0.82-2.82	
Positive EAT-10		(27) 8.48	5.71-11.25	
	Male	(9) 8.22	4.36-12.08	0.6722
	Female	(18) 8.61	4.66-12.55	
RSI (Total)		(160) 1.56	3.30-5.15	
	Male	(68) 3.50	2.31-4.69	0.2416
	Female	(92) 4.76	3.40-6.12	
Positive RSI		(13) 19.62	15.70-23.53	
	Male	(4) 17.5	10.09-24.91	0.1920
	Female	(9) 20.55	15.03-26.07	

Both total and positive only EAT-10 scores were significantly correlated to RSI and regarding gender-specific correlations this was evident in females but not males. No significant correlations were found of EAT-10 or RSI with age or gender. Linear regression revealed a trend towards the decent of the EAT-10 score with age progression. However, this was non-significant. Mann Whitney non-parametric test for investigating differences between means showed no differences between genders regarding the RSI or the EAT-10. Again, these were analyzed both with all subjects included and with only the positive responders ( Table 2 ).

**Table 2:** Correlations between genders in regards to the RSI and the EAT-10

		Pearson's coefficient r	P- value
EAT-10/RSI	Total	0.565	**<0.001**
	Male	0.195	0.112
	Female	0.701	**<0.001**
Pos EAT-10 / Pos RSI	Total	0.849	**0.016**
	Male	0.805	0.053
	Female	0.834	**0.039**

## Discussion

The European Society for Swallowing Disorders and the European Union of Geriatric Medicine Society have recognized Dysphagia as a geriatric syndrome. 2 Most studies for the estimation of the prevalence of dysphagia among elderly are conducted within nursing homes or other institutions. 1 3 6 7 The definition of dysphagia among them shows a great variation, as does the perception of dysphagia among the questioned individuals. That is partly why the prevalence of dysphagia has shown a wide distribution over the years and among different countries. In the largest epidemiological study in nursing homes across 19 different countries, a single question was asked to the local nursing home staff, whether the resident had dysphagia or not. 7 Prevalence was recorded with a range from 4 to 48% in different countries.

Using Videoflouroscopy, Ekberg in one of the most cited early studies reported that from the 56 studied individuals without known dysphagia, only 9 (16%) showed normal swallowing function. 1 This early paper includes primarily individuals with different types of disease that with our today's knowledge can hardly be regarded as “normal swallowers”, including dementia patients and patients with Zenker's diverticulum. A recent population-based study report that the overall prevalence of self-reported swallowing difficulties in Canadian adults over the age of 45 was 10.6% and increased with age progression. 17 Our findings suggest a correlation between self-reported dysphagia and LPR symptoms; however, this relationship should be interpreted with caution due to the absence of objective diagnostic tests. Subclinical dysphagia—where swallowing dysfunction exists without overt symptoms—may be underdiagnosed in our study, as it requires instrumental assessments like videofluoroscopy or fiberoptic endoscopic evaluation of swallowing (FEES). Studies have shown that aging-related sensory changes and silent aspiration can lead to underreported dysphagia, potentially influencing our results. 18 Nevertheless, most authors today agree that dysphagia among the elderly ranges around 30–40%. 19

The disparity in prevalence rates might be due to the different national health systems or private facilities and the ability or training of the caregivers to recognize the symptoms or report them. This is why self-reported dysphagia with validated questionnaires is possibly more accurate.

The community-dwelling elderly population has been left out by many studies. This is an important information gap in our understanding of the actual prevalence of dysphagia in the elderly. Even in the few studies where this population was addressed, it actually included individuals requiring long-term care and receiving home medical care, such as nursing, nutrition, and rehabilitation. 20 A recent survey from Japan by using the EAT-10 that was sent by postal mail found a prevalence of 25.1% (128/510) among independent individuals, and percentages differed significantly among age groups ( *P* < 0.001). This figure was significantly higher (53.8%) in the very general group of “dependent” individuals.

In various countries like Greece, most of the aging population is community-dwelling due to social habits, family bonds and lack of high-quality state-run facilities. This has been evident during the last decade partly due to the austerity-hit health and social support system.

The EAT-10 questionnaire has a high sensitivity and is applicable to different languages and eating habits. 13 21 It has been proven to detect aspiration in patients with neurologic diseases that often reside undiagnosed within the elderly population. For these reasons it has been recommended as a first-line tool for systematic screening 21 and has been proposed as an indicator of the nutritional status of the elderly. 20

The aging population has multiple comorbidities such as dementia, movement disorders, the history of stroke and is also subject to polypharmacy. All of these situations are independent factors that lead to dysphagia and some of them have been indeed underemphasized, such as the impact of multiple medications on swallowing.

It seems that in half of our 16.47% of individuals that were found to have swallowing disorders, LPR could be the primary cause of their symptoms. It is shown that dysphagia is significantly correlated with LPR, even though the RSI is probably not the most sensitive screening tool to diagnose LRP in these individuals. 22 It is also shown that with aging, symptoms of reflux are less prominent despite a tendency of older patients toward greater severity of esophageal mucosal injury. 15 This means that we could make the assumption that an additional unknown number of dysphagia patients in our elderly population could be attributed to reflux. It thus seems possible that dysphagia is more related to reflux than other mechanisms attributed to frailty and the aging upper alimentary tract.

Our prevalence of dysphagia remains lower than most other papers where community-dwelling population was studied. It is comparable with one study from Atlanta, Georgia. By using the M.D. Anderson Dysphagia Inventory (MDADI) and the general health Short Form-12 survey (SF-12v2 ^TM^ ) in an urban senior independent-living community, the authors reported a 15.9% prevalence of dysphagia, and a 15% when using a single question whether the individual had or not swallowing difficulties. This was the first study to question the initial findings of Lindgren and Janzon who reported a 35% prevalence of dysphagia among an urban population aged 50- to 75-year-old. 23 The reported prevalence was based upon at least one positive answer to questions attributed to various swallowing symptoms including and not limited to globus and gastroesophageal reflux.

On the other hand, the MDADI was also used by Roy et al in the USA and showed a prevalence of 32.5% of independent seniors living in Utah and Kentucky. Serra-Prat et al used the Volume-Viscosity Swallow Test (V-VST) to estimate what they regarded as the “real” prevalence of dysphagia. 24 The test uses fusing boluses of different volumes and viscosities, administered in a progression of increasing difficulty and is also validated but is implemented by “trained general practitioners” and thus cannot be used as a screening tool.

### Limitation

This study presents several limitations that should be acknowledged. First, the reliance solely on self-reported questionnaires (EAT-10 and RSI) limits the ability to confirm objective findings of dysphagia and LPR. While these tools are validated and widely used for screening, they may lack the precision of diagnostic tests such as videofluoroscopy or fiberoptic endoscopic evaluation of swallowing in identifying subclinical dysphagia. Consequently, the prevalence of dysphagia in our study may be underestimated, as individuals with subtle or asymptomatic swallowing dysfunctions may not have been detected. Future research should incorporate objective diagnostic methods to provide a more comprehensive assessment of dysphagia prevalence and severity in community-dwelling elderly populations.

## Conclusion

This is one of the few studies that examine the possible association between geriatric dysphagia and reflux in a rural population. A 23.0% prevalence of dysphagia was observed in elderly adults (70 years or older), as well as a trend towards a decent increase in both EAT-10 and RSI scores with age progression. Dysphagia is prevalent among the elderly, but there are considerable discrepancies between studies in regard to methodology and outcomes. Social habits, community support and other country-specific differences such as dietary habits might influence these results. The RSI and EAT-10 questionnaires remain reliable screening tools for geriatric LPR and dysphagia that can be used by health care professionals. More studies are needed to explore this field.
